# Supplementary figures and images for: Microbial and small zooplankton communities predict density of baleen whales in the southern California Current Ecosystem
Source: PLoS One. 2026 May 6;21(5):e0334209. doi: 10.1371/journal.pone.0334209 (PMC13148692; doi:10.1371/journal.pone.0334209)

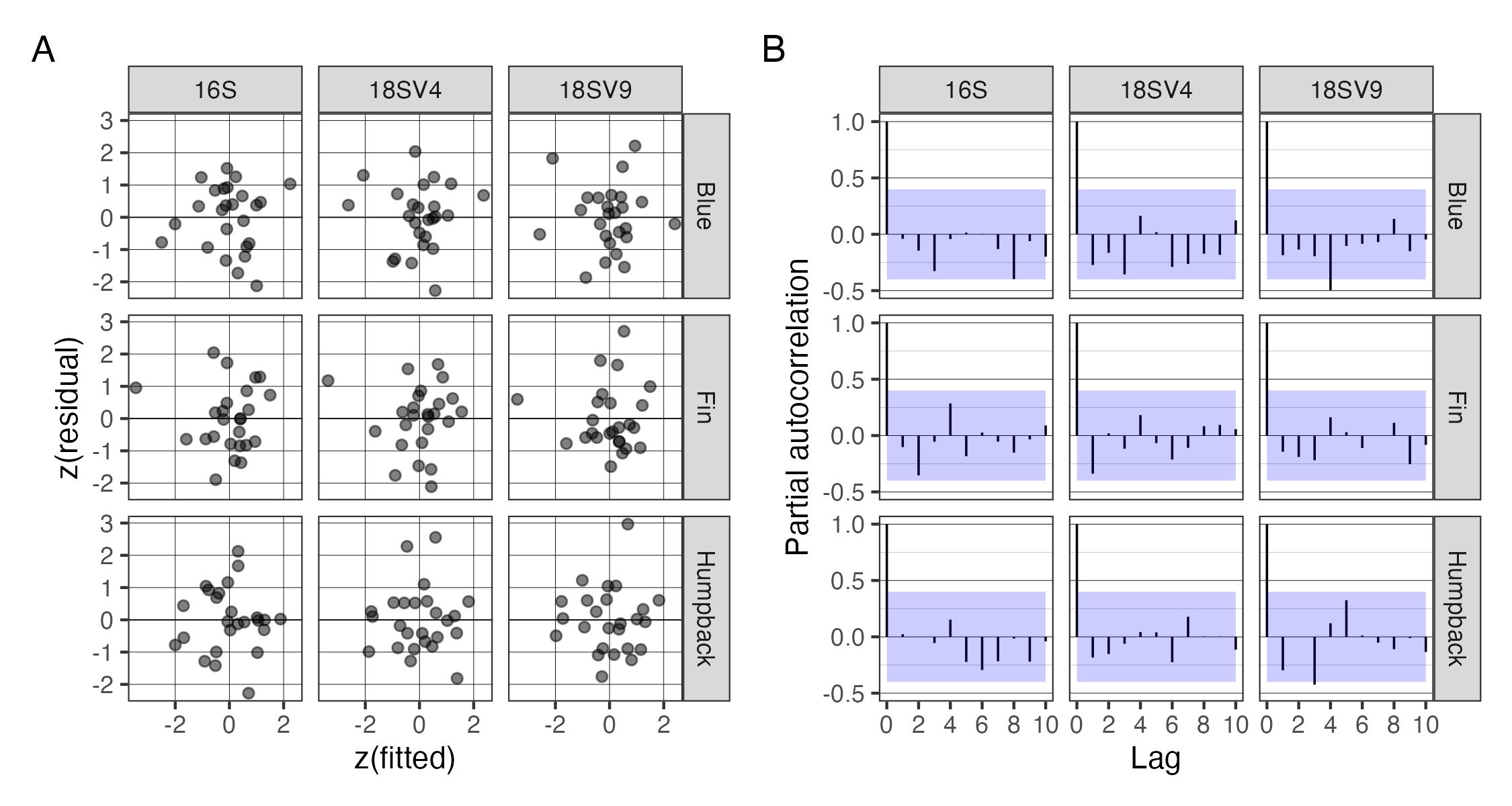

Supplement: S1 Fig — A) residuals against fitted values for each model (whale species and marker combination) and B) partial autocorrelations for each model to assess possible time dependence across successive surveys not accounted for in the models. (TIFF) [file pone.0334209.s006.tiff]
