# Supplementary material for: Microbial and small zooplankton communities predict density of baleen whales in the southern California Current Ecosystem
Source: PLoS One. 2026 May 6;21(5):e0334209. doi: 10.1371/journal.pone.0334209 (PMC13148692; doi:10.1371/journal.pone.0334209)
Supplement: Attachment — Submitted filename: PONE-D-25-51982_Review_Final.pdf [file pone.0334209.s007.pdf]

PONE-D-25-51982

Microbial and small zooplankton communities predict density of baleen whales in the southern California Current Ecosystem

This study presents an innovative and rigorous approach to predicting baleen whale population densities using microbial and small plankton communities as ecological indicators. The concept of assessing microbial assemblages (via eDNA metabarcoding) to infer the distribution and/or abundance of higher trophic species (cetaceans, elasmobranchs, etc) is highly relevant and aligns with current advances in molecular ecology and ecosystem modeling. Overall, the paper offers an impressive integration of visual sightings data metabarcoding, and statistical modeling, and it makes a valuable contribution to marine eDNA research as well as cetacean ecology.

However, several sections of the manuscript would benefit from further clarification and reorganization to strengthen its overall coherence, especially for a broad journal audience like that of PLOS. The Introduction section requires more context and flow to clearly establish the study rationale, objectives, and novelty. Concepts that are critical to the study, such as eDNA, metabarcoding, and the ecological framing of “microbial habitats”, should be introduced earlier and defined clearly to guide readers who may be unfamiliar with these concepts. The Methods section, while generally sound, needs substantially more detail on laboratory procedures, particularly regarding contamination avoidance measures, sample handling, and sequencing protocols, to ensure transparency. The statistical analyses are a clear strength of the paper. The modeling framework is sophisticated and very well-explained. The results are also presented nicely and highlight meaningful ecological relationships across trophic levels.

The Discussion, while well-written, could go further in connecting the findings to broader ecological and conservation implications. Including a “future outlook” section that considers the applications of this predictive framework, for instance, in whale population monitoring and management, would enhance the manuscript’s impact.

In summary, this is a well-designed study that contributes greatly to the understanding of whale-microbe ecological linkages. With a more cohesive introduction, expanded methodological detail, and a stronger framing of broader applications, the manuscript will be suitable for publication. Please also address my line-by-line feedback below.

ABSTRACT

- Line 14: Would you be able to change the wording in the first sentence? As it is worded, "density of marine mammals" sounds like the framework for a physiological paper.
- Lines 15-18: Consider removing the parenthetical phrase "– the potential 'ecological habitat' –" to improve sentence clarity and flow. It feels redundant given the context and interrupts the main idea.
- Line 20: can you please elaborate on "each quarterly season" or change the wording?
- Line 21: Which densities were estimated?
- Lines 24-25: please include the words "environmental DNA" in this sentence.
- Line 26: You do not need to provide an acronym for ASVs if it is not used again in the abstract (saves word count).
- Lines 28-30: what do you mean by "naive seasonal carry-forward and seasonal averaging approaches to prediction"? I'm not sure that this is the correct phrasing… please change or remove this.
- I suggest you check word count for the journal abstract, this seems a bit long and it's generally unusual to have two paragraphs in an abstract.
- You may want to add "metabarcoding" to the keywords and remove "quantitative ecology".

INTRODUCTION

- Line 52: Provide cultural value to whom or what?
- The first paragraph should flow a bit more cohesively. This can be achieved by including a sentence that connects the conservation/management idea with the three study species and then another sentence which connects the study species to your study area. As it is written, the California Current is suddenly introduced without a purpose.
- Line 63: I suggest not using the word "track", as the whales are not directly "tracking" the krill species. Perhaps instead use "and are correlated with present aggregations".
- Beware of introducing new species throughout your manuscript – there is currently an unsystematic pattern of using Latin vs common names.
- Line 77: Do you mean habitat-specific ecology?
- Line 78: The work is not particularly novel - there are many studies linking microbes/plankton presence and density to whale presence (e.g., Friedlaender et al., 2006; Fielder et al., 2018; Pendleton et al., 2012). You could rewrite this to indicate that the novel approach is based on metabarcoding data, but this has still been done before… (e.g., Carroll et al., 2019; Visser et al., 2021; Boyse et al., 2024). If this study is truly novel, please describe how in more detail to convince readers.
- Line 83: A brief definition of microbiome (pertaining to whale biology) may be useful for readers of this journal (not particularly marine scientists/cetacean researchers).
- Line 105: Please include some references for this.
- Line 108: Again, why the California Current Ecosystem? I think the study site should be introduced briefly here. Is it a hot spot for baleen whale activity?

- Lines 109-111: You just contradicted the information that you have written thus far, if it has been challenging, how is it a novel approach and/or how are you justifying the means for this study? I suggest removing this sentence.
- Line 112: eDNA and/or metabarcoding hasn't been introduced! This is a key component of this study and needs to be described in detail, especially as this journal is for non-specialists.
- Lines 113-115: This needs references.
- Lines 119-121: This also needs references.
- Line 124: Please add a meaningful concluding sentence for the end of the introduction (e.g., future outlooks, how this study can effectively promote better methods of researching whales).

METHODS

- Lines 127-128: This is information that was missing in the introduction to justify the study site and its importance.
- Line 144: What do you mean by "mean = 3.3 L", were there different water volumes collected? Was that on purpose?
- Line 146: Companies and products should be included with a reference and category number.
- How were the materials cleaned between samples to avoid cross-contamination?
- Where and how was labwork conducted? How were surfaces disinfected/cleaned to avoid contamination?
- Lines 147-148: Again, please reference the company and cat # (this comment persists throughout the rest of the methods).
- Line 149: Were all samples included in the libraries? Or just positive detections based on bands from the gels?
- Line 159: You have already described gels earlier in the methods section but it makes more sense here. Please remove the first instance.
- Line 161: What do you mean by PCR quantification?
- Lines 171-172: What about the NextSeq run?
- In general, please be very specific about how the data were assessed regarding markers - you could even include supplementary information regarding the overlap of ASVs detected across markers.
- Lines 202-206: This needs references.
- Line 238: Was that to reduce potential contamination effects?
- Lines 287-290: Can you include 1-2 more references for this?
- Line 358: You are now double-justifying that this approach of correlating whales with microbes/plankton has been taken before, indicating that the "novel" approach that you've written in the introduction is not true.

RESULTS

- Line 397: "fairly uniformly" is not proper for a results section as it's quite ambiguous. Please rephrase.
- Line 431: Again, "highly sparse" is subjective.

- Line 462: Again “strongly predictive”.
- Line 469: “yielded” would be a better word for “gave” here.

DISCUSSION

- Line 515-517: Here is the sentence that explains the novelty of this work that sets it apart from the other whale/prey/symbiote association papers. I suggest rewording the information that you’ve given in the introduction and discussion to match this syntax.
- Line 562: The strain level?
- Line 595: I think this should be an em dash not an en dash.
- Line 598-600: This makes me think, how were the primers selected? Please include 1-2 sentences on this in the methods. Please also include some references for this sentence.
- Line 602: Again, please include more references.
- Line 603: Please be careful again with the dashes.

FIGURES

- Figure 1: Please add a north arrow to your maps.
- Figure 2: Can you please include units on your y-axis label?
- Figure 3: Again, please include units on the y-axis for density.

TABLES

- No comments.

SUPPLEMENTARY INFORMATION

Very nice layout of your spreadsheets! The data you provided here are useful and easy to interpret.
