# Supplementary material for: Microbial and small zooplankton communities predict density of baleen whales in the southern California Current Ecosystem
Source: PLoS One. 2026 May 6;21(5):e0334209. doi: 10.1371/journal.pone.0334209 (PMC13148692; doi:10.1371/journal.pone.0334209)
Supplement: Attachment — Submitted filename: FINAL_response-to-reviewers_PLOSONE.docx [file pone.0334209.s009.docx]

Response to reviewers

PONE-D-25-51982

*We would like to thank you and the reviewers for their thoughtful review, which has substantially improved our manuscript. We appreciate the time and effort you took to review our work, and we have carefully addressed all the comments and suggestions. Our responses are provided in italicized font below and highlighted via track changes in the attached document. All references to lines below refer to the new document line numbers (with track changes).*

*Thank you!*

Journal Requirements

When submitting your revision, we need you to address these additional requirements.

1. Please ensure that your manuscript meets PLOS ONE's style requirements, including those for file naming.

***We have updated the manuscript formatting following the provided guides and implemented the following changes:***

- ***Remove numbering and adjust Level 1 and Level 2 headers to sentence case in 18pt and 16pt font size, respectively***
- ***Format figure and table titles in bold and add legends for figures and tables appearing in the body of the paper***
- ***Change figure citations from Figure 1, Figure 2, etc., to Fig 1, Fig 2, etc.***
- ***Change supplementary figure/table citations to S1 Fig, S1 Table, etc.***
- ***Update author byline symbol for equal contributions and corresponding author***
- ***Add corresponding author email***
- ***Add departments to author affiliations where missing***

***We are holding on updating author-year citations to numbered citations pending any further revisions requested by reviewers that may alter the number and order of appearance of references.***

2. Thank you for stating the following financial disclosure:

“This material is based upon research supported by the Office of Naval Research under Award Number (N00014-22-1-2719), upon work supported by National Oceanic and Atmospheric Administration grants NA15OAR4320071 and NA19NOS4780181 and Simons Foundation Collaboration on Principles of Microbial Ecosystems (PriME) grant 970820. EVS was supported by a partnership among CalCOFI participants, including Scripps Institution of Oceanography (SIO), NOAA Southwest Fisheries Science Center (NA20OAR4170258), California Department of Fish and Wildlife (#P2370002), and California Sea Grant (NA22OAR4170106).”

Please state what role the funders took in the study. If the funders had no role, please state: "The funders had no role in study design, data collection and analysis, decision to publish, or preparation of the manuscript."

If this statement is not correct you must amend it as needed.

Please include this amended Role of Funder statement in your cover letter; we will change the online submission form on your behalf.

***We have included the suggested statement regarding funders’ role: "The funders did not have a role in study design, data collection and analysis, decision to publish, or preparation of the manuscript." We also would like to add “This research is partly based upon work supported by the Research, Scholarly & Creative Activities Program awarded by the Cal Poly Division of Research.”***

***The complete funding statement should read “This material is based upon research supported by the Office of Naval Research under Award Number (N00014-22-1-2719), upon work supported by National Oceanic and Atmospheric Administration grants NA15OAR4320071 and NA19NOS4780181 and Simons Foundation Collaboration on Principles of Microbial Ecosystems (PriME) grant 970820. EVS was supported by a partnership among CalCOFI participants, including Scripps Institution of Oceanography (SIO), NOAA Southwest Fisheries Science Center (NA20OAR4170258), California Department of Fish and Wildlife (#P2370002), and California Sea Grant (NA22OAR4170106). This research is partly based upon work supported by the Research, Scholarly & Creative Activities Program awarded by the Cal Poly Division of Research. The funders did not have a role in study design, data collection and analysis, decision to publish, or preparation of the manuscript.”***

3. When completing the data availability statement of the submission form, you indicated that you will make your data available on acceptance. We strongly recommend all authors decide on a data sharing plan before acceptance, as the process can be lengthy and hold up publication timelines. Please note that, though access restrictions are acceptable now, your entire data will need to be made freely accessible if your manuscript is accepted for publication. This policy applies to all data except where public deposition would breach compliance with the protocol approved by your research ethics board. If you are unable to adhere to our open data policy, please kindly revise your statement to explain your reasoning and we will seek the editor's input on an exemption. Please be assured that, once you have provided your new statement, the assessment of your exemption will not hold up the peer review process.

***Data are currently publicly available at*** [***https://doi.org/10.5281/zenodo.15678927***](https://doi.org/10.5281/zenodo.15678927)***; we have amended the data availability statement accordingly.***

4. If the reviewer comments include a recommendation to cite specific previously published works, please review and evaluate these publications to determine whether they are relevant and should be cited. There is no requirement to cite these works unless the editor has indicated otherwise.

***No reviewer comments included a recommendation to cite specific previously published works.***

Reviewer #1

The study uses a dataset from the CalCOFI monitoring program to model relative abundances of bacteria and small plankton inferred from metabarcoding with baleen whale observations (here blue, fin, and humpback) to understand if assessing microbial and small plankton communities can be used for predicting baleen whale densities. Overall, the study is robust, and I only had a few questions on the methods and how some choices might influence the overall model results. A very interesting approach with potential application to other systems (e.g. could we use this for fish? Other marine mammals?), but I wonder how well it will perform under longer time scales or other regions. The authors do a fantastic job of discussing the challenges of this approach and the need for further research to validate their findings.

***Thank you. We have integrated the idea expressed in the comment: “A very interesting approach with potential application to other systems (e.g. could we use this for fish? Other marine mammals?), but I wonder how well it will perform under longer time scales or other regions” into the concluding sentence (lines*** ***1108-1113)***.

Line 30 – forgive my ignorance, but what is RMSE? Consider defining here as you have done with ASV on line 26

***We have replaced the acronym with “root mean square prediction error”.***

Line 158 –curious if 56 deg C was used for the 16S? the Parada set typically uses an annealing temp closer to 50 deg C and wonder how this might impact observed biodiversity and subsequent patterns and results.

***The annealing temperature depends on MgCl_2_ concentrations in PCR buffer which differ between our study and that used by Parada et al. (2016). In accordance with the higher salt concentration in our PCR buffer, our annealing temperature is higher. The master mix used by Parada et al. (2016) recommends a minimum annealing temperature of 50°C, whereas ours recommends a minimum of 57°C; thus, we believe that our annealing temperature of 56°C is appropriately similar, while being high enough to minimize off-target amplification.***

182 – In considering the method used to count cetaceans, I completely understand the reasoning for using passing mode (as explained in Campbell et al.) but also note that they acknowledge that it results in “more unidentified or mis-identified groups, more biased estimates of group size, and less precise species percentages than closing mode”. How might this impact marine mammal density estimates and subsequently the results presented? The authors do a fantastic job of accounting for other biases introduced by the data (e.g. seasonal influence, metabarcoding) in their model but I wonder if this is accounted for as well. Certainly counting marine mammals at sea is difficult, so I appreciate the level of scrutiny the authors impose, just more curious about this than anything.

***Because we did not correct for the percentage of unidentified animals among those sighted in the surveys, our estimates likely underestimate the true density. However, the relative seasonal estimates and contributions of each species wouldn't change that much if unidentified detection rates are similar across species.***

Reviewer #2

This study presents an innovative and rigorous approach to predicting baleen whale population densities using microbial and small plankton communities as ecological indicators. The concept of assessing microbial assemblages (via eDNA metabarcoding) to infer the distribution and/or abundance of higher trophic species (cetaceans, elasmobranchs, etc) is highly relevant and aligns with current advances in molecular ecology and ecosystem modeling. Overall, the paper offers an impressive integration of visual sightings data metabarcoding, and statistical modeling, and it makes a valuable contribution to marine eDNA research as well as cetacean ecology.

However, several sections of the manuscript would benefit from further clarification and reorganization to strengthen its overall coherence, especially for a broad journal audience like that of PLOS.

The Introduction section requires more context and flow to clearly establish the study rationale, objectives, and novelty. Concepts that are critical to the study, such as eDNA, metabarcoding, and the ecological framing of “microbial habitats”, should be introduced earlier and defined clearly to guide readers who may be unfamiliar with these concepts.

***We have substantially revised the Introduction to establish the study rationale and objectives, clarify the novelty, and introduce key concepts (e.g., eDNA/metabarcoding, and the ecological habitat concept) (lines 242-252; 282-313; and 426-463).***

The Methods section, while generally sound, needs substantially more detail on laboratory procedures, particularly regarding contamination avoidance measures, sample handling, and sequencing protocols, to ensure transparency.

***We have substantially revised the Methods section to include more detail on laboratory procedures (lines 490-520), particularly regarding contamination avoidance measures (lines 495-496), sample handling (lines 490-493), and sequencing protocols (lines 514-520).***

The statistical analyses are a clear strength of the paper. The modeling framework is sophisticated and very well-explained. The results are also presented nicely and highlight meaningful ecological relationships across trophic levels.

The Discussion, while well-written, could go further in connecting the findings to broader ecological and conservation implications. Including a “future outlook” section that considers the applications of this predictive framework, for instance, in whale population monitoring and management, would enhance the manuscript’s impact.

***We have substantially revised the text in the Discussion to connect the findings to broader applications and implications of the work. Please see lines 1072 – 1113.***

In summary, this is a well-designed study that contributes greatly to the understanding of whale-microbe ecological linkages. With a more cohesive introduction, expanded methodological detail, and a stronger framing of broader applications, the manuscript will be suitable for publication. Please also address my line-by-line feedback attached to this review.

***Thank you. We have addressed the broader comments related to the introduction, expanded methodological detail, and a stronger framing of broader applications above and in the line by line comments below.***

ABSTRACT

● Line 14: Would you be able to change the wording in the first sentence? As it is worded, “density of marine mammals” sounds like the framework for a physiological paper.

***We changed to distribution and abundance.***

● Lines 15-18: Consider removing the parenthetical phrase “– the potential ‘ecological habitat’ –” to improve sentence clarity and flow. It feels redundant given the context and interrupts the main idea.

***Removed.***

● Line 20: can you please elaborate on “each quarterly season” or change the wording?

***We have changed this to “each season”.***

● Line 21: Which densities were estimated?

***We have clarified by adding “Densities of Balaenopteridae whales”.***

● Lines 24-25: please include the words “environmental DNA” in this sentence.

***We have included “environmental DNA” in the sentence to read “We identified microbial and small plankton communities from environmental DNA that were specific to each target species and were strong predictors of estimated density”.***

● Line 26: You do not need to provide an acronym for ASVs if it is not used again in the abstract (saves word count).

***We have removed the acronym as suggested.***

● Lines 28-30: what do you mean by “naive seasonal carry-forward and seasonal averaging approaches to prediction”? I’m not sure that this is the correct phrasing… please change or remove this.

***We have revised this sentence to improve clarity, replacing it with: “Our approach improves out-of-sample root mean square prediction error by up to 65% compared with simple alternative methods.”***

● I suggest you check word count for the journal abstract, this seems a bit long and it’s generally unusual to have two paragraphs in an abstract.

***Thank you for catching this. We have shortened the abstract to less than 300 words (as per guidance by the PLOS One submission guidelines).***

● You may want to add “metabarcoding” to the keywords and remove “quantitative

Ecology”.

***We have removed “quantitative ecology” and added “metabarcoding” to the keywords.***

INTRODUCTION

● Line 52: Provide cultural value to whom or what?

***We added “to people” since aesthetic, spiritual, educational, and recreational experiences contribute to their cultural value for human societies.***

● The first paragraph should flow a bit more cohesively. This can be achieved by including a sentence that connects the conservation/management idea with the three study species and then another sentence which connects the study species to your study area. As it is written, the California Current is suddenly introduced without a purpose.

***We have substantially edited the Introduction so that it flows from the conservation management idea to the three species and study area. Please see lines 242 – 252; 253-284; 426 – 428.***

● Line 63: I suggest not using the word “track”, as the whales are not directly “tracking” the krill species. Perhaps instead use “and are correlated with present aggregations”.

***We edited to “their distributions tend to be correlated with krill aggregations” since they are most likely following the krill or something that is very closely associated with them (likely an acoustic or chemical signature).***

● Beware of introducing new species throughout your manuscript – there is currently an unsystematic pattern of using Latin vs common names.

***We follow the general convention of using the full common name followed by the scientific name at first mention (e.g., “Blue whale (Balaenoptera musculus)”). After first mention, we use common names (e.g., “blue whales”). The only exception to this convention involves taxa that do not have a well-known common name like Thysanoessa spinifera. For these species, we provide only the scientific names.***

● Line 77: Do you mean habitat-specific ecology?

***Yes. We have changed “biological” to “ecological”.***

● Line 78: The work is not particularly novel - there are many studies linking microbes/plankton presence and density to whale presence (e.g., Friedlaender et al., 2006; Fielder et al., 2018; Pendleton et al., 2012). You could rewrite this to indicate that the novel approach is based on metabarcoding data, but this has still been done before... (e.g., Carroll et al., 2019; Visser et al., 2021; Boyse et al., 2024). If this study is truly novel, please describe how in more detail to convince readers

***We have clarified that our approach builds on these existing studies and efforts to assess the ability of the entire planktonic community to predict the density of baleen whales in the California Current. The benefit of our study lies in the breadth of taxa sampled in the water column and the spatial and temporal scale of our analyses. We have clarified this in Introduction (see lines 306 – 313) and in the Discussion (lines 1073-1078).***

● Line 83: A brief definition of microbiome (pertaining to whale biology) may be useful for readers of this journal (not particularly marine scientists/cetacean researchers).

***Added a brief definition of microbiome – “which consists of communities of microorganisms living on and in the whale”.***

● Line 105: Please include some references for this.

***We have included references to support this sentence.***

● Line 108: Again, why the California Current Ecosystem? I think the study site should be introduced briefly here. Is it a hot spot for baleen whale activity?

***We have included a description of the study system and the relevance and importance of it (lines 426-428).***

● Lines 109-111: You just contradicted the information that you have written thus far, if it has been challenging, how is it a novel approach and/or how are you justifying the means for this study? I suggest removing this sentence.

***We removed this text and have clarified the unique value of this study in the Introduction (see lines 306 – 313) and in the Discussion (lines 1073-1078).***

● Line 112: eDNA and/or metabarcoding hasn’t been introduced! This is a key component of this study and needs to be described in detail, especially as this journal is for non-specialists.

***We added text to introduce the concept of eDNA. “This technique captures DNA from single-celled microbes and cells shed by eukaryotic multicellular organisms into the water column to generate a molecular “fingerprint” of the biological community spanning multiple trophic levels”. Please see lines 433-435.***

● Lines 113-115: This needs references.

***We have added relevant references.***

● Lines 119-121: This also needs references.

***We have added relevant references.***

● Line 124: Please add a meaningful concluding sentence for the end of the introduction (e.g., future outlooks, how this study can effectively promote better methods of researching whales).

***We have added a sentence to conclude the introduction that emphasizes the potential of our approach to inform future research and modeling efforts linking microbial communities to baleen whale ecology. Please see lines 458-463.***

METHODS

● Lines 127-128: This is information that was missing in the introduction to justify the study site and its importance.

***We added the importance of the study site into the introduction (see comment above).***

● Line 144: What do you mean by “mean = 3.3 L”, were there different water volumes collected? Was that on purpose?

***Yes, different volumes were collected. The nature of field work and collecting hundreds of samples over many cruises makes it impossible for all volumes to be identical. We now clarify this by removing that text and stating, “the average volume filtered was 3.3 L.”***

● Line 146: Companies and products should be included with a reference and category number.

***We have added company names and catalog numbers where appropriate.***

● How were the materials cleaned between samples to avoid cross-contamination?

***We now describe that the bottles and lines used for sampling were rinsed with Milli-Q water and the sample itself prior to collection (lines 490-493).***

● Where and how was labwork conducted? How were surfaces disinfected/cleaned to avoid contamination?

***We now state that lab work was performed at the J. Craig Venter Institute with ethanol-cleaned tools and workspaces. We also now describe how we included extraction blanks and PCR negative controls to confirm the absence of contamination throughout the process. We believe that the revised manuscript now describes the methods with sufficient detail to enable replication (lines 484- 533).***

● Lines 147-148: Again, please reference the company and cat # (this comment persists throughout the rest of the methods).

***We have added company names and catalog numbers where appropriate.***

● Line 149: Were all samples included in the libraries? Or just positive detections based on bands from the gels?

***Yes, all samples were included, and this is now described in the methods. A lack of amplification is extremely rare within this dataset and is usually associated with a sample that had known issues during collection or preservation. Those samples are excluded from the final analysis (lines 515 – 518).***

● Line 159: You have already described gels earlier in the methods section but it makes more sense here. Please remove the first instance.

***We respectfully disagree with the reviewer’s suggestion. The first gel confirms that we have genomic DNA from our sample immediately after extraction. The second gel confirms that we have PCR product of the correct size for the amplicon of interest. As a result, we believe that to be as detailed as possible in our methods, it is necessary to include both instances.***

● Line 161: What do you mean by PCR quantification?

***We have revised this to now state “DNA quantification of the PCR product” (line 514).***

● Lines 171-172: What about the NextSeq run?

***We have removed “MiSeq” to specify that this sentence applies to all runs. Thank you for catching this error.***

● In general, please be very specific about how the data were assessed regarding markers - you could even include supplementary information regarding the overlap of ASVs detected across markers.

***With the revisions, we believe that the methods text are sufficiently detailed to enable replication (lines 484- 533). We are unsure what the reviewer is referring to with respect to overlap of ASVs. ASVs represent unique DNA sequences and as we are sequencing different genes or regions, there is inherently no overlap. As a result, we have not made additions to the supplementary information in response to this comment.***

● Lines 202-206: This needs references.

***We added a reference to Roberts et al. (2016), which shows the importance of accounting for variation in detectability when making inferences about cetacean habitat associations (lines 565-566).***

● Line 238: Was that to reduce potential contamination effects?

***The rationale was that these ASVs would be uncorrelated with marine mammal density (or any other variable) owing to their minimal variability in the study region. We have revised this sentence to further clarify: “We removed rare ASVs present in under 1% of samples across all cruises and abundant ASVs present in more than 99% of samples across all cruises, as neither rare nor ubiquitous ASVs yield data with sufficient variation to provide a basis for prediction of marine mammal density.” (lines 599-601).***

● Lines 287-290: Can you include 1-2 more references for this?

***We included additional references we believe would be most useful for our audience: a broadly accessible review paper (Abdi 2010) and a reference on application of PLS in ecology (Carrascal et al. 2009). However, there is an extensive literature on PLS and if the reviewer wants, e.g., classic works, recent books/reviews, or any other specific reference type, we are happy to include these as well.***

● Line 358: You are now double-justifying that this approach of correlating whales with microbes/plankton has been taken before, indicating that the “novel” approach that you’ve written in the introduction is not true.

***As mentioned above, we have now clarified the unique value of our work in the Introduction (see lines 306 – 313) and in the Discussion (lines 1073-1078).***

RESULTS

● Line 397: “fairly uniformly” is not proper for a results section as it’s quite ambiguous. Please rephrase.

***While we appreciate that “fairly” evokes ambiguity, in this instance sampling was not exactly uniform, so some modifier is necessary for accuracy of expression. We have replaced the adverb with “approximately”, which perhaps more clearly conveys the distinction we wish to draw here.***

● Line 431: Again, “highly sparse” is subjective.

***We have removed the adverb and provided a numeric measure of sparsity.***

● Line 462: Again “strongly predictive”.

***We have removed the adverb.***

● Line 469: “yielded” would be a better word for “gave” here.

***We have adopted the suggestion.***

DISCUSSION

● Line 515-517: Here is the sentence that explains the novelty of this work that sets it apart from the other whale/prey/symbiote association papers. I suggest rewording the information that you’ve given in the introduction and discussion to match this syntax.

***As mentioned above, we have now clarified the unique value of our work in the Introduction (see lines 306 – 313) and in the Discussion (lines 1073-1078).***

● Line 562: The strain level?

***We have reworded to clarify “Marine microbial diversity is vast and marker gene sequences are not reliable indicators of fine-scale taxonomy or ecological niche” (lines 1012-1014).***

● Line 595: I think this should be an em dash not an en dash.

***We thank the reviewer for their attention to detail and adjusted.***

● Line 598-600: This makes me think, how were the primers selected? Please include 1-2 sentences on this in the methods. Please also include some references for this Sentence.

***We now state in the methods that “The primers used here were established by previous studies to capture the entire microbial community with commonly-used marker gene regions, and have been shown to outperform other primer sets (McNichol et al. 2015).” See lines 499 – 501.***

● Line 602: Again, please include more references.

***We have added papers from the statistical literature on variable selection via regularization that discuss conditions leading to selection instability.***

● Line 603: Please be careful again with the dashes.

***We thank the reviewer for their attention to detail and have adjusted.***

FIGURES

● Figure 1: Please add a north arrow to your maps.

***We have added a north arrow to each map. We do note that the lat/lon axes clearly convey cardinal directions in the original figure.***

● Figure 2: Can you please include units on your y-axis label?

***We have added “individuals per 1000 km^2^” parenthetically to the axis label.***

● Figure 3: Again, please include units on the y-axis for density.

***We have added “individuals per 1000 km^2^” parenthetically to the axis label.***

TABLES

● No comments.

SUPPLEMENTARY INFORMATION

Very nice layout of your spreadsheets! The data you provided here are useful and easy to interpret.
