# Supplementary material for: Microbial and small zooplankton communities predict density of baleen whales in the southern California Current Ecosystem
Source: PLoS One. 2026 May 6;21(5):e0334209. doi: 10.1371/journal.pone.0334209 (PMC13148692; doi:10.1371/journal.pone.0334209)
Supplement: Attachment — Submitted filename: PONE-D-25-51982R1_response.docx [file pone.0334209.s010.docx]

Dr. Erin Satterthwaite

Scripps Institution of Oceanography, UCSD

8622 Kennel Way

La Jolla, CA 92037

esatterthwaite@ucsd.edu

Dr. Vitor Hugo Rodrigues Paiva

PLOS

1875 Mission Street

Suite 103 #188

San Francisco, CA 94103

*March 2, 2026*

Re: PONE-D-25-51982R1, *Microbial and small zooplankton communities predict density of baleen whales in the southern California Current Ecosystem*

Dear Dr. Paiva,

Thank you for the invitation to revise and resubmit our manuscript. We have revised the manuscript in response to the reviewers’ comments and believe we have addressed the remaining concerns. Below, we provide a detailed response to each comment, and we attach copies of the revised manuscript with and without tracked changes.

We appreciate your continued consideration of our manuscript for publication in PLOS One.

We also note that “The funders had no role in study design, data collection and analysis, decision to publish, or preparation of the manuscript” so we request that this be added to our online submission.

Sincerely,

Dr. Erin Satterthwaite & Dr. Trevor Ruiz

**Reviewer #2**

I thank the authors for their thorough and thoughtful revision of their manuscript. I have only a few minor comments for consideration before the final version is published.

ABSTRACT

Line 24-28: The microbes/plankton communities are not directly predicting whale density, rather, they may influence whale behavior and distribution, with the prediction performed by the modeling approach applied in the study.

***We agree that microbial and plankton communities are not directly determining whale density. However, we use “predict” in the statistical sense and we are careful to avoid causal language throughout the abstract. Here at the outset we prefer to retain “predict” rather than add qualifiers that might obscure the sentence, but have added a clarification to the fourth sentence of the abstract that we intend statistical rather than causal or direct prediction.***

Line 32: “in” rather than “using”

***We have adopted the suggestion.***

Line 38: Please add a comma between “parasite” and “or” to follow your sentence structure.

***We have adopted the suggestion.***

INTRODUCTION

I’m sorry! I am still wary of the first sentence as it’s the first instance in which you can draw in readers and establish credibility for the study. At first, you state that larger baleen whales play a vital role in marine ecosystems, but the supporting point for this introductory statement is that they “provide cultural value to people…” which is not a role in a marine ecosystem, rather an anthropogenic effect of whales. Please change this sentence.

***We have rearranged the sentence to make clear that we are making separate statements about the significance of whales in ecological, cultural, and conservation contexts. It now reads, “Large baleen whales play a vital role in marine ecosystems by helping to regulate ecosystem processes [1]. They are of conservation and management relevance, as any populations are listed as threatened (Vulnerable, Endangered, or Critically Endangered) on the IUCN Red List of Threatened Species [2], and they provide significant cultural value to people [3].”***

Lines 88-94: I think this is a better place (rather than lines 111-113) to introduce eDNA as you are introducing metabarcoding here (given that the journal audience may not know about eDNA and/or metabarcoding).

***We prefer to retain the current narrative structure, but have revised this sentence to remove the term “metabarcoding” and replaced with “molecular methods” so that it is not introduced before it is defined clearly.***

METHODS

Lines 153-157: Is it possible to state how often these work spaces were cleaned and at which percentage was the ethanol?

***We used 70% ethanol and cleaned the workspace before each use.***

Line 172: Which ratio of beads:product was used?

***We have clarified that 1x was used.***

Line 176: Okay, I see 0.8x was used, perhaps you could also state this above.

***As noted above we have revised as suggested.***

Line 184: In my initial comment here, I asked about overlapping ASVs. I had meant overlapping species (and/or genus, family) assignments (from ASVs) that were identified with both markers. For example, it would be interesting to know how many of x plankton species (or genus) were identified across each gene of interest. However, this is not critical to the outcomes of this paper and is not necessary to be included.

***Since the line reference pertains to the molecular methods rather than analysis, we feel that this information would be more appropriate to include in the Results section and have added a brief comment at the first mention of S2 Table containing taxonomic annotations. We are careful to clarify in this addition that the overlap percentages reported are among candidates used in the models, i.e., after relative abundance filtering, with the implication that the percentages reported should not be viewed as accurate estimates of overlap among all detections across markers. We feel that the post-filtering overlap is more salient to the analysis in the paper than the pre-filtering overlap. That said, however, the same summary statistics among annotations prior to filtering are 12.8%, 3.9%, and 44.7% (compared with 9.9%, 0.7%, 51.2%), so while the filtering evidently introduces some bias to the overlap measures, it is not so substantial as to alter the relative interpretation of the extent of overlap across markers.***

RESULTS

No further comments.

DISCUSSION

Line 542: “strong” would be a better word than “good” here.

***We have adopted the suggestion.***

Lines 549-556: It is not usual to include further numerical results and references to tables in the discussion. I see that PLOS does have flexibility for a “Results and Discussion” section, but as you have kept them separate, I am wondering if some of the information in this paragraph should be kept in the Results section rather than repeated here. I don’t think there is a “good” or “bad” way to go about this, and if you and your co-authors agree that the data should be kept in this part of the discussion, then that is okay, but I figured that I would note this. 4

***We appreciate the note and agree that the discussion reads more clearly without the two sentences that reiterate the quantitative findings of the paper. These have been removed.***

Lines 590-592: This statement may read a bit strong as marker genes can still be informative at various taxonomic scales. You may want to slightly de-escalate the wording to emphasize limitations in fine-scale taxonomic/ecological resolution rather than unreliability. The sentence could be rephrased: Marine microbial diversity is vast, and while marker-gene amplicons (e.g. 16S or 18S rRNA) are valuable for broad community characterization, they often provide insufficient resolution to infer fine-scale taxonomy or ecological niche (83-85).

***We have adopted the suggestion.***

FIGURES

A small note regarding the north arrow and lat/long: I did not mean to come off as condescending here - as someone with a degree in GIS, north arrows were one of the “requirements” that I was trained to include in any map(s) that are generated for publication. I do agree that latitude and longitude denote coordinate location sufficiently for readers, and upon reading up on this, it seems that over the past years, north arrows are no longer seen as one of the four key elements (scale bar, north arrow, legend, coordinate grid) of maps. So thank you for the learning opportunity and I will keep this in mind as I review other papers!

***We appreciate the clarification and were not aware that this was a strong convention among geographers or those with GIS training, so the reviewer’s comment serves as a learning opportunity for us as well.***

**Reviewer #3**

General comments

From reviewing the response to the previous reviewer comments, the authors have done a good job incorporating the feedback into this version of the manuscript. I have therefore provided my review on the ‘revised manuscript with tracked changes’ version.

Overall, the manuscript is well-written, interesting, and scientifically sound. I have made a couple of very minor comments below. Congrats to the authors on such a thorough, impactful study!

Specific comments

Lines 70-71: May be best to capitalize listing status (check journal requirements however)

***We have amended the sentence to include the exact listing categories that correspond to “threatened” parenthetically and have capitalized those categories.***

Lines 218-220: Is there a reason you didn’t use bleach (or similar) to fully decontaminate sampling bottles? As you didn’t collect sampling blanks, it is hard to determine if any species for sure were not carried over between the sampling events

***It was not feasible to use bleach or another cleaning agent in between each collection event. We also now clarify that the bottles were rinsed with the sample three times before sampling. The Milli-Q rinse likely not only removed cells from the wash itself, but also from osmotic shock as these are marine organisms. Further rinsing with the sample before filling provides the opportunity to remove any remaining organisms from the bottle. Even if something remained in the bottle, its abundance relative to the sample, which was multiple liters of seawater, certainly was drastically reduced.***
